# Supplementary material for: Reduced antibody cross-reactivity following infection with B.1.1.7 than with parental SARS-CoV-2 strains
Source: eLife. 2021 Jul 29;10:e69317. doi: 10.7554/eLife.69317 (PMC8352583; doi:10.7554/eLife.69317)
Supplement: Supplementary file 1. — This table lists the number, median age (and range), gender proportion, and the median time (and range) post infection for the donors and patients studied. [file elife-69317-supp1.docx]

**Table S1. Donor and patient characteristics.**

| **Donor/patient group** | **Number** | **Age, years (range)** | **Male gender (%)** | **Days post infection^2^, median (range)** |
| --- | --- | --- | --- | --- |
| Acute D614G infection, mild/asymptomatic^1^ | 17 | 32  (26-66) | 7  (41) | 28  (6-35) |
| Acute D614G infection, COVID-19 patients | 20 | 61  (25-73) | 14  (70) | 25  (14-43) |
| Acute B.1.1.7 infection, mild/asymptomatic | 29 | 57  (20-99) | 18  (62) | 11  (4-46) |

^1^Seroconversion and/or first positive RT-qPCR in the first month of enrolment (month 1). The same group of participants were samples again 2 months later (month 3).

^2^Days post symptom onset for symptomatic cases and days post first positive RT-qPCR test for asymptomatic cases.
